# Influence of Varroa Mite (*Varroa destructor*) Management Practices on Insecticide Sensitivity in the Honey Bee (*Apis mellifera*)

**DOI:** 10.3390/insects8010009

**Published:** 2017-01-11

**Authors:** Frank D. Rinkevich, Robert G. Danka, Kristen B. Healy

**Affiliations:** 1 Honey Bee Breeding, Genetics, and Physiology Laboratory, Agricultural Research Service, USDA, Baton Rouge, LA 70820, USA; Bob.Danka@ars.usda.gov; 2 Department of Entomology, Life Sciences Annex, Louisiana State University, Baton Rouge, LA 70803, USA; KHealy@agcenter.lsu.edu

**Keywords:** honeybee, Varroa mite, insecticide sensitivity, amitraz

## Abstract

Since Varroa mites may cause devastating losses of honey bees through direct feeding, transmitting diseases, and increasing pathogen susceptibility, chemical and mechanical practices commonly are used to reduce mite infestation. While miticide applications are typically the most consistent and efficacious Varroa mite management method, miticide-induced insecticide synergism in honey bees, and the evolution of resistance in Varroa mites are reasonable concerns. We treated colonies with the miticide amitraz (Apivar^®^), used IPM practices, or left some colonies untreated, and then measured the effect of different levels of mite infestations on the sensitivity of bees to phenothrin, amitraz, and clothianidin. Sensitivity to all insecticides varied throughout the year among and within treatment groups. Clothianidin sensitivity decreased with increasing mite levels, but no such correlation was seen with phenothrin or amitraz. These results show that insecticide sensitivity is dynamic throughout the 5 months test. In-hive amitraz treatment according to the labeled use did not synergize sensitivity to the pesticides tested and this should alleviate concern over potential synergistic effects. Since IPM practices were largely ineffective at reducing Varroa mite infestation, reliance on chemical methods of Varroa mite management is likely to continue. However, miticides must be used judiciously so the long term effectiveness of these compounds can be maximized. These data demonstrate the complex and dynamic variables that contribute to honey bee colony health. The results underscore the importance of controlling for as many of these variables as possible in order to accurately determine the effects of each of these factors as they act alone or in concert with others.

## 1. Introduction

The Varroa mite, *Varroa destructor*, is one of the most important causes of colony declines and increased overwintering colony losses in the honey bee, *Apis mellifera*. Varroa mites feed on the hemolymph of pupae and adults, which can result in premature mortality. The impacts of Varroa mite infestation can be immediate and profound. In some regions of the US, up to 80% of managed colonies were lost due to Varroa mite infestation in the 1995–1996 field season [1]. Varroa mite levels as low as 10 mites per 100 bees can reduce overwintering survival [2].

While the direct damage to honey bees by Varroa mites is evident, mite infestation also indirectly increases the susceptibility to other parasites and diseases. For example, low to moderate Varroa mite infestations can reduce the expression of antimicrobial peptides, dampen immunity function, facilitate virus amplification, and may affect the expression of genes related to behavior [3,4,5]. High mite infestation can lower pupal and adult weight [6,7], which can lead to lower reproductive output by drones as well as reduced colony maintenance and foraging capabilities by workers.

Varroa mite infestation affects physiological processes that are relevant to insecticide sensitivity. Varroa mite infestation can reduce body size [6,7], and body size is a universal factor that dictates sensitivity to insecticides. Varroa mites may affect insecticide sensitivity through lowering the titer of vitellogenin in the hemolymph of infested bees [8]. Vitellogenin is a carrier protein that can act to sequester xenobiotics and limit oxidative stress [9], and high vitellogenin levels may account for the different acaricide sensitivities between workers and queens [10]. Varroa infestation dampens expression of genes involved in metabolic detoxification and oxidative stress [11]. Due to its effects on body size, vitellogenin titers, and metabolic gene expression, it is reasonable to conclude that Varroa mite infestation may increase insecticide sensitivity.

Many control methods are used to reduce Varroa mite populations. Chemical control is often the most effective and most economical control measure. However, Varroa mites have evolved resistance to fluvalinate [12], coumaphos [13], and amitraz [14]. Decreased miticide sensitivity leads to over application of these miticides, resulting in high concentrations of these chemicals found in honey bee colonies [15]. Synergistic interactions between miticides and insecticides have been demonstrated [16,17], so their impact on colony health is of concern. Alternatively, Integrated Pest Management (IPM) strategies for Varroa mite can be implemented, which may include screened bottom boards [18], drone brood trapping [19], and grooming stimulation via powdered sugar application [20]. However, the effectiveness of each treatment is variable and inconsistent [21,22,23].

The goals of this study were to evaluate (1) if bees infested with high levels of Varroa mites are more sensitive to insecticides than bees with lower levels of mites; (2) if Varroa mite management practices affect insecticide sensitivity; and (3) if Varroa mite management practices are equally effective.

## 2. Materials and Methods

### 2.1. Seasonal Management Experiments

Thirty-six colonies of Italian honey bees (Wooten’s Golden Bees, Palo Cedro, CA, USA) were established in single-story deep Langstroth hives on 4 May 2015 at the USDA-ARS (United States Department of Agriculture, Agricultural Research Service) Honey Bee Breeding, Genetics, and Physiology Laboratory in Baton Rouge, LA, USA, under normal field rearing conditions. No Varroa mite treatments, antibiotics, or supplemental feedings were administered beyond the scope of this research. Boxes of frames with wax-coated foundations were added based on the needs of the colony. The colonies were divided into three treatment groups (control, amitraz, and IPM) so that the initial Varroa mite infestations were equal among groups. Varroa mites were not managed in the control group. The amitraz group received treatments in the form of Apivar^®^ strips (Veto-Pharma, Mauritius, France) according to the label instructions (i.e., one strip per five combs with brood). Treatments were applied from 13 May 2015 through 7 July 2015 and from 1 September 2015 through the end of the experiment. Varroa mite levels in the IPM group were managed using non-chemical control methods of screened bottom boards, trapping Varroa mites in drone brood, and coating bees with powdered sugar to dislodge Varroa mites. Screened bottom boards (Better Bee, Greenwich, NY, USA) were installed at the colony establishment on 4 May 2015 and remained in place through the duration of the experiment. One full drone comb was placed in each colony at colony establishment. The drone comb was removed and frozen when sealed drone brood was present and replaced with an empty drone comb. Bees were treated with powdered sugar (Great Value, Bentonville, AR, USA) by removing frames and coating bees with an even layer of powdered sugar by shaking powdered sugar through a screened shaker. Powdered sugar treatments were administered 6 May 2015, 8 July 2015, and 2 September 2015.

Varroa mite infestation was measured by sampling approximately 300 bees from the brood-bearing comb; these samples were then stored at −20 °C until processed. Varroa mites were dislodged from bees by shaking in warm soapy water at 120 rpm on an orbital table shaker for 1 h and then were counted. Samples were reshaken until no additional Varroa mites were dislodged. The bees were counted and Varroa infestation was calculated by dividing the total number of mites by the number of bees in the sample and converting to number of mites per 100 bees.

### 2.2. Pesticides

We evaluated the sensitivity of honey bees to the insecticides phenothrin and clothianidin and the miticide amitraz. Phenothrin is an active ingredient in insecticide formulations used in mosquito control programs. Clothianidin is used as a seed treatment in many industrially grown crops, such as corn and soy. Amitraz is formulated as Apivar^®^ and is used on impregnated strips as an in-hive chemical treatment to control Varroa mites. All materials were >98% purity and were purchased from ChemService (West Chester, PA, USA). Stock solutions of each compound were dissolved in acetone.

### 2.3. Bioassays

Bioassays were performed as previously described [17]. Brood frames were collected from each colony the first Monday of each month (May through October) and held at 33 ± 1 °C with 70% ± 1% humidity in a dark incubator. One-day-old bees were brushed from the frames and sorted into groups of 20 in disposable wax paper cups, which were covered with tulle, secured with a rubber band, and held at the environmental conditions listed above with three cotton balls soaked in 50% (w/v) sucrose solution until bees were three-days of age.

Stock solutions of pesticides were diluted to include four to seven concentrations that provided more than 0% and less than 100% mortality. Phenothrin and amitraz stock solutions were diluted in acetone, while clothianidin was diluted in 50% (w/v) sucrose solution. The concentrations of phenothrin used in the bioassays were 100, 75, 66, 50, 33, 25, and 10 ng/uL. Amitraz concentrations were 12,500, 7500, 5000, 2500, 1250, 750, and 500 ng/uL. Topical bioassays with phenothrin and amitraz were performed by applying a 1 uL drop of insecticide with a mechanical Hamilton syringe to the thoracic notum of a bee anesthetized on CO_2_ for less than 1 min. Control topical bioassays were performed with 1 uL of acetone. The 20 anesthetized bees were weighed after treatment. Bees were held at the environmental conditions listed above. Feeding bioassays with clothianidin were performed by placing a perforated microcentrifuge tube with filled with 1 mL of clothianidin in 50% sucrose solution through the tulle covering the waxed cup and removing the cotton ball soaked with sucrose solution. The concentrations of clothianidin used in bioassays were 1.0, 0.75, 0.5, 0.25, 0.1, 0.075, and 0.05 ng/uL. Control feeding assays were conducted with 50% sucrose solution with 0.001% acetone. At least three replications of 20 bees per cup were used at each dose for each pesticide; this was used to determine LD_50_/LC_50_ values with a minimum of 200 bees per pesticide per treatment group. Mortality in all bioassays was recorded at 24 h after insecticide application. Individuals that were ataxic or unable to right themselves were scored as dead.

### 2.4. Statistical Analyses

The LD_50_/LC_50_ value for each insecticide for each colony was calculated using probit analysis with Abbot’s correction for control mortality [24] and standardized by body weight using Minitab (Minitab, State College, PA, USA). Toxicity was considered significantly different if the 95% CI of the LD_50_/LC_50_ values did not overlap between treatments or test dates. All other statistics were performed with JMP 12 (SAS, Cary, NC, USA). The amitraz LD_50_ and Varroa infestation levels were log-transformed to establish a normal distribution that was tested with the Shapiro-Wilk W Test. Varroa mite infestation and bee weight among treatments over time were compared by One-Way ANOVA. The relationship between pesticide sensitivity, Varroa infestation, and body weight were analyzed by linear regression.

## 3. Results

### 3.1. Varroa Infestation

**Figure 1:**
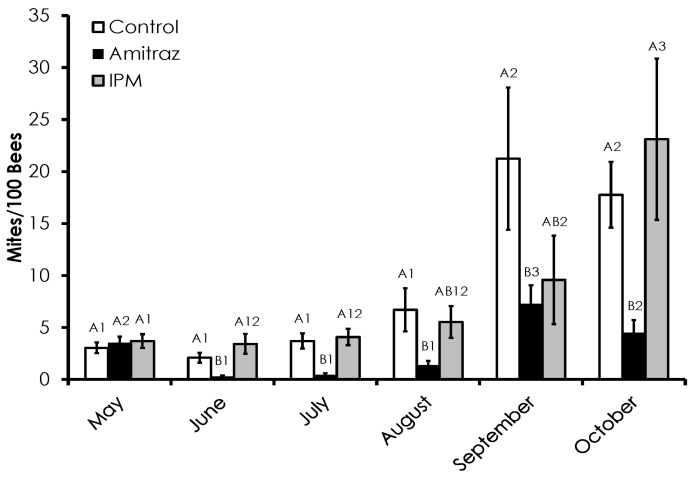
Varroa mite infestation levels over time among the Control, Amitraz, and IPM groups. Amitraz treatments in the form of Apivar^®^ strips were administered twice for 56 days beginning on 13 May 2015 and 1 September 2015. Data are shown as the average ± SEM. Bars with different letters within the same sampling date indicate significant differences between treatment groups at that sampling date. Bars with different numbers indicate significant differences within treatment groups over sampling dates.

Varroa mite infestation rates were variable among treatment groups, but the amitraz treated group was consistently the lowest (Figure 1). Despite starting with equal Varroa mite infestation levels, Varroa mite infestation was significantly lower in the amitraz treated colonies compared to the control and IPM colonies in June (*F* = 6.61, *p* = 0.004), July (*F* = 9.65, *p* = 0.001), and October (*F* = 4.6, *p* = 0.02). Amitraz treated colonies had lower Varroa mite infestation levels than the control group, but not the IPM group in August (*F* = 3.43, *p* = 0.05) and September (*F* = 3.89, *p* = 0.05). Varroa mite infestation levels increased throughout the duration in all treatment groups. The control (*F* = 7.2, *p* << 0.001) and amitraz treated colonies (*F* = 11.31, *p* << 0.001) had mite levels in September and October that were higher than all of the preceding months. Varroa infestation was higher in September than October in the amitraz treated group. The IPM group Varroa infestation level in October was significantly higher than the preceding months and the September infestation level was significantly higher than in May (*F* = 6.62, *p* << 0.001). The pattern and rate of increase in Varroa mite infestation levels were different among treatments. The pattern of mite growth in the control group was exponential and significant (R^2^ = 0.63, *p* < 0.0001). A linear relationship was seen in the amitraz treated group, but the relationship was poor and insignificant (R^2^ = 0.27, *p* = 0.28). Varroa infestation in the IPM group increased in an exponential manner and was highly significant (R^2^ = 0.95, *p* = 0.0009). The rate of increase in Varroa mite infestation in amitraz treated colonies was significantly lower than in the control (*df* = 8, *t* = 2.39, *p* = 0.044) and IPM colonies (*df* = 8, *t* = 2.33, *p* = 0.047).

### 3.2. Honey Bee Weight

**Figure 2:**
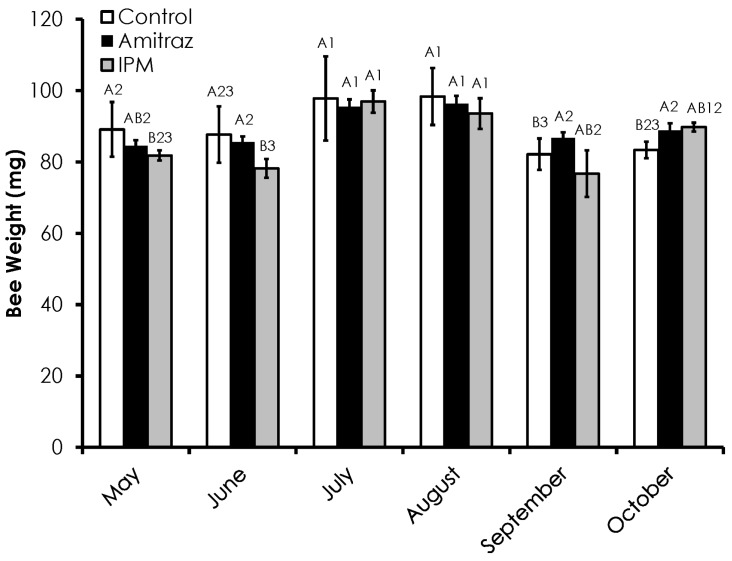
Adult honey bee weight over time among the Control, Amitraz, and IPM groups. Data are shown as the average ± SEM. Significant differences between and within treatment groups is as described in Figure 1.

Honey bee weight varied among treatments and through time (Figure 2). Honey bee weight in the IPM colonies was significantly lower than in the control colonies in May (*F* = 6.19, *p* = 0.024), but similar to both the control and amitraz colonies in June (*F* = 7.91, *p* = 0.001). In September (*F* = 5.46, *p* = 0.026) and October (*F* = 4.57, *p* = 0.05), honey bee weight in the control colonies was significantly lower compared to the amitraz treated colonies. Honey bee weight peaked in July and August in all treatments. Bee weight in the control colonies was highest in July and August, while bee weight in May, June, and October was not significantly different, as was bee weight in June, September and October.

### 3.3. Bioassays

Bioassays showed that the sensitivities of bees to insecticides varied among mite treatment groups and within treatment groups over time. Phenothrin sensitivity was equal among all treatments in May and July (Table 1). Bees in the IPM treatment group had significantly higher phenothrin sensitivity in June compared to the control and amitraz treatments. In August, both the control and IPM treatments had higher phenothrin sensitivity than the amitraz treatment. However, the amitraz treated bees were more sensitive to phenothrin in September and October than were the control. Within the control group, phenothrin sensitivity in any month was not significantly different from the initial sensitivity evaluated in May. The highest phenothrin sensitivity in the control group in August was significantly different than all other months besides May, while the lowest sensitivity was seen in July and September. Phenothrin sensitivity in the amitraz treated group was significantly higher in October compared to any other month. Furthermore, phenothrin sensitivity in the amitraz treated group was significantly higher in June compared to May and August. Phenothrin sensitivity was highest in the IPM group in June, and it was significantly different compared to July, August, and September. August’s LD_50_ value was significantly different compared to June, July, and September.

Sensitivity to amitraz varied with no consistent pattern among and between treatment groups (Table 2). In May, amitraz sensitivity was higher in the control and amitraz groups compared to the IPM group. Amitraz sensitivity was highest in the amitraz treated group in June compared to the control and IPM groups. The IPM group was more sensitive to amitraz than the amitraz treated group in August. In September, amitraz sensitivity was highest in the amitraz and IPM treated colonies. The control group was more sensitive to amitraz than the amitraz treated group in October and the LD_50_ for amitraz was unable to be calculated from the IPM group in October.

Clothianidin sensitivity was significantly higher in the control and amitraz group than the IPM group in May (Table 3). The IPM group was more than 11-fold more sensitive to clothianidin than the control group in June. The IPM group was more sensitive to clothianidin than the amitraz group in August. In September, the amitraz group was more sensitive to clothianidin than the control group. The amitraz and IPM groups were more sensitive to clothianidin than the control group in October. Within the control group, clothianidin sensitivity was highest in May, June, and July, intermediate in August and October, and lowest in September. Clothianidin sensitivity in the amitraz group was highest in May and June, which was significantly different from July, which was significantly different from October, which was significantly different from August and September. The IPM group had the highest sensitivity to clothianidin in June while the lowest sensitivity was in May and September.

### 3.4. Interactions of Varroa Infestation, Pesticide Sensitivity, and Weight

**Figure 3:**
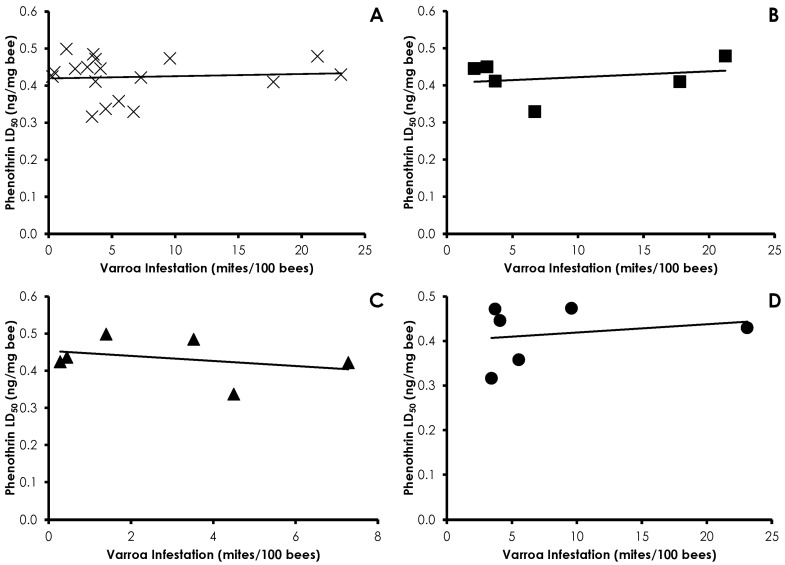
Influence of Varroa infestation on phenothrin sensitivity in all treatments (**A**); Control (**B**); Amitraz (**C**); and IPM (**D**) treatment groups. There was no significant effect of Varroa infestation on phenothrin sensitivity in any instance.

**Figure 4:**
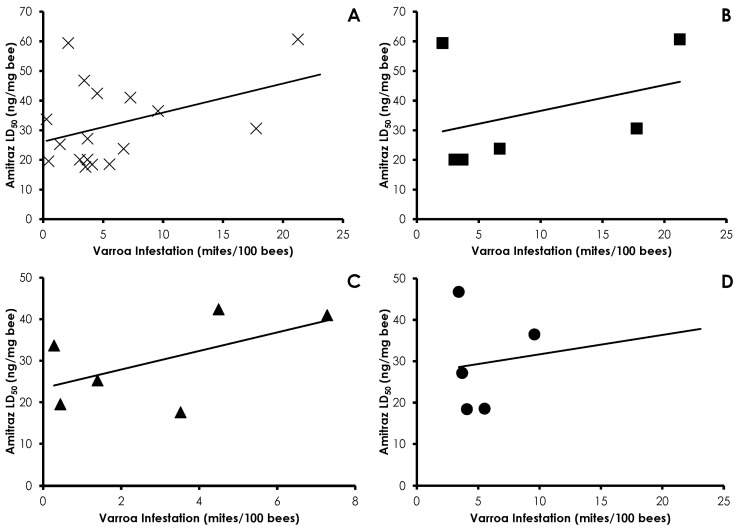
Influence of Varroa infestation on amitraz sensitivity in all treatments (**A**); Control (**B**); Amitraz (**C**); and IPM (**D**) treatment groups. There was no significant effect of Varroa infestation on amitraz sensitivity in any instance.

**Figure 5:**
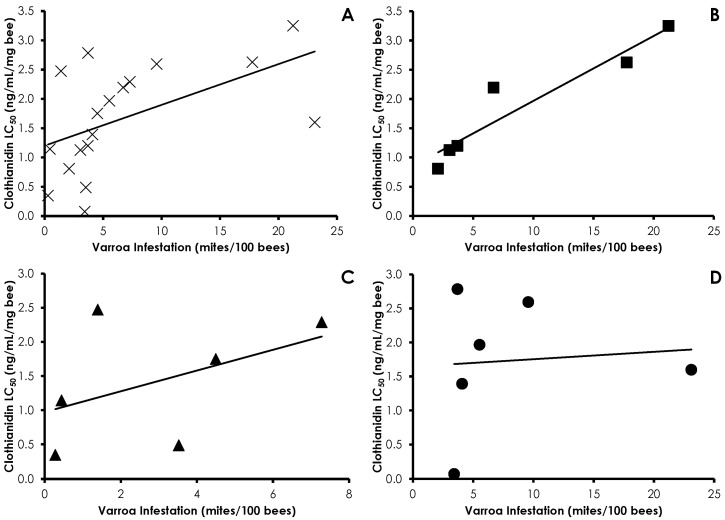
Influence of Varroa infestation on clothianidin sensitivity in all treatments (**A**); Control (**B**); Amitraz (**C**); and IPM (**D**) treatment groups. Clothianidin sensitivity significantly decreased with increasing mite infestation in all treatments and in the control group.

Varroa infestation did not affect phenothrin sensitivity in honey bees (*df* = 17, *F* = 0.087, *p =* 0.77, R^2^ = 0.005), and there were no treatment effects (Control *df* = 5, *F* = 0.003, *p =* 0.95, R^2^ = 0.0007; Amitraz *df* = 5, *F* = 0.167, *p =* 0.70, R^2^ = 0.0006; IPM *df* = 5, *F* = 0.340, *p =* 0.59, R^2^ = 0.001; Figure 3). A similar lack of correlation was found with amitraz sensitivity and Varroa infestation in all cases (*df* = 16, *F* = 0.930, *p =* 0.35, R^2^ = 0.05) and within treatments (Control *df* = 5, *F* = 0.293, *p =* 0.61, R^2^ = 0.06; Amitraz *df* = 5, *F* = 0.419, *p =* 0.55, R^2^ = 0.09; IPM *df* = 4, *F* = 0.004, *p =* 0.95, R^2^ = 0.001; Figure 4). Clothianidin sensitivity significantly decreased with increasing mite infestation levels (*df* = 17, *F* = 7.76, *p =* 0.013, R^2^ = 0.32; Figure 5). The effect was seen in the control treatment (*df* = 5, *F* = 94.17, *p =* 0.0006, R^2^ = 0.95), but not the amitraz treatment (*df* = 5, *F* = 1.33, *p =* 0.31, R^2^ = 0.249) or the IPM treatment (*df* = 5, *F* = 0.197, *p =* 0.68, R^2^ = 0.047).

**Figure 6:**
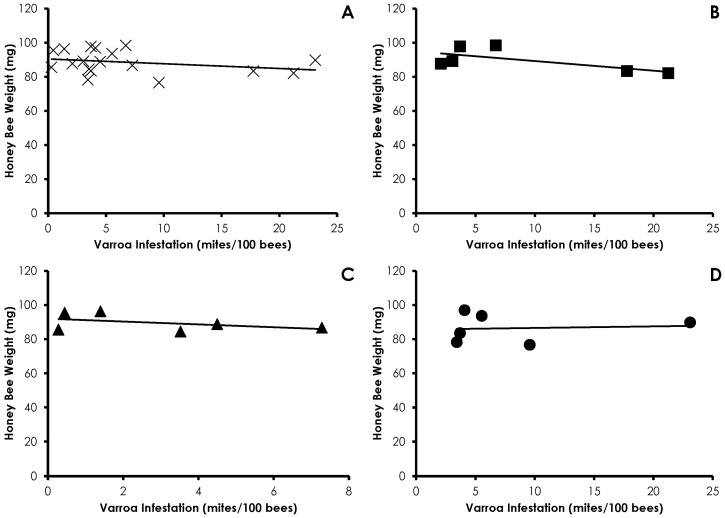
Influence of Varroa infestation on honey bee weight in all treatments (**A**); Control (**B**); Amitraz (**C**); and IPM (**D**) treatment groups. There was no significant effect of Varroa infestation on honey bee weight in any instance.

Honey bee weight was not affected by Varroa infestation (*df* = 17, *F* = 1.15, *p =* 0.29, R^2^ = 0.06) and there was no treatment effect (Control *df* = 5, *F* = 1.35, *p =* 0.30, R^2^ = 0.253; Amitraz *df* = 5, *F* = 0.460, *p =* 0.53, R^2^ = 0.103; IPM *df* = 5, *F* = 0.0075, *p =* 0.93, R^2^ = 0.001; Figure 6).

## 4. Discussion

Honey bee colony health is a complex and dynamic manifestation of an increasingly nuanced summation of biotic and abiotic factors [25]. Understanding the interactions of these factors that promote colony health is of the utmost importance to the commercial pollination industry in the US that provides nearly $20B in direct and indirect crop value [26].

While our seasonal mite management experiments were terminated in October, we originally planned to continue them through December. However, the control and IPM colonies in October displayed a high frequency of overt symptoms of deformed wing virus (DWV) and chronic bee paralysis virus (CBPV), presumably due to high mite infestation in those treatment groups. Our concern was that, although viral titers were not measured, the presumed high prevalence of infection leading to poor adult emergence and high control mortality would have confounded our bioassay results, especially in the case of amitraz bioassays in the IPM group in October. We chose not to use any colonies that had a high prevalence of virus-mediated symptoms. Future experiments to assess insecticide sensitivity in bees with known virus infection rates will determine possible interactions of these factors.

There was no evidence of amitraz synergism in our bioassays using field colonies, despite synergism being documented in laboratory studies [16,17]. Phenothrin sensitivity in the amitraz group was the same as in the control or IPM group throughout the experiment. A similar lack of consistent amitraz synergism was observed in amitraz bioassays. While clothianidin sensitivity was higher in the amitraz group compared to the control group in September and October, clothianidin sensitivity was also higher in the IPM group compared to the control in those same two months. Taken together, this shows that amitraz treatment did not synergize pesticide sensitivity. The cost of potential amitraz synergism may be small relative to the benefit of reduced Varroa infestation.

The differences in Varroa mite infestation levels between treatments showed that amitraz has a significant impact on mite populations compared to control and IPM treatments. The control and IPM colonies reached Varroa mite infestation levels by September of 21.2 and 9.5 Varroa mites/100 bees, respectively. Such infestations often lead to colony death over winter [27,28]. The screened bottom boards and powdered sugar treatments administered in the IPM treated group were not adequate to suppress mite levels below those in colonies with no control measures. IPM treatment did slow the rate of Varroa mite infestation in August and September to the point where it was not statistically different from the amitraz treatment. However, the IPM treatment did not stop the dramatic increase in Varroa mite infestation so that in October the IPM treatment produced the highest Varroa mite infestation levels seen in the experiment. These findings are largely in line with previous reports that IPM measures provide limited effectiveness at controlling Varroa mite populations at the colony level through the season [29,30]. Drone brood trapping was likely not very effective in our treatment scheme because very little drone brood (<200 capped drone cells/comb) was present at any time that it was removed from the colony. Other studies of drone brood trapping in reducing Varroa mite infestation levels were effective when large numbers of drone brood were removed (>7000 capped drone cells [19], >3000 cells [31]). The lack of consistently efficacious and easily administered IPM techniques contributes to the reliance on chemical control of Varroa mites. It is likely that Varroa mite suppression by amitraz treatment would have been enhanced if treatment were been continuous throughout the year. The gap between amitraz treatments (to mimic a honey harvest) allowed for the mite population to rebound dramatically. For practical purposes concerning managing mites with chemical means, the beekeeper may have to balance taking a honey crop with colony survival. The current Apivar^®^ label limits application to 2 treatments annually with a 56 days maximum treatment interval. Amendment of the Apivar^®^ label to allow uninterrupted year-round treatment would very likely improve product effectiveness in the short term. However, a constant treatment regime would also increase selection pressure for amitraz resistance in Varroa mites. The loss of effective amitraz treatments to control Varroa mites is a disconcerting prospect due to the low rate of product development to specifically and effectively control Varroa mites.

The lack of detrimental effects of Varroa infestation on pesticide sensitivity was unexpected, as it is well documented that Varroa infestation has physiological aspects that are relevant to pesticide sensitivity [6,7,8,11]. Decreased clothianidin sensitivity with increased mite infestation was especially confounding. Varroa infestation peaks in the fall, which may coincide with seasonal physiological changes. For example, seasonal differences in pesticide sensitivity have been demonstrated in the case of reduced diazinon sensitivity in fall, which was correlated with increased cytochrome-P450 activity [32]. However, for this explanation to be accurate, the changes in pesticide sensitivity would be consistent across all treatment groups; for clothianidin as well as with phenothrin [17]. Another consideration is that we did not directly measure Varroa infestation on bees used in bioassays. Perhaps there is a disparity between sampling Varroa infestation at the colony level and measuring pesticide sensitivity at the individual level. Future work on the interaction of Varroa infestation and pesticides should be performed by measuring Varroa infestation on bees using bioassays.

We found that honey bee weight was not influenced by mite infestation, despite previous reports of such an interaction [6,7]. This incongruence likely arises from the different methods used to measure mite infestation. Our study used mite infestation rates determined from mites on workers collected from brood comb and weight from workers with unknown infestation levels, while other studies measured mite infestation rate in brood pupal cells and directly weighed those newly emerged adults. Therefore, employing a Varroa sampling measure that more intimately connects it with other measures of honey bee health may more accurately assess those interactions.

## 5. Conclusions

This study highlights how many real world practices can affect insecticide sensitivity. These results underscore the difficulty in comparing results of honey bee toxicology from study to study, due to the difficulty in controlling for all these variables. This study and many other studies show that Varroa mite infestation is a major factor affecting colony health. The fact that amitraz strips were a significantly more effective method of controlling Varroa mites than the IPM measures we implemented suggests that amitraz will continue to be used intensely in at least the short term. Use of this product is likely to lead to a higher prevalence of amitraz-resistant Varroa mites [14], thus reducing the effectiveness of amitraz as a mite management tool. Use of Varroa-resistant bees (i.e., Varroa-Sensitive Hygienic (VSH) bees) [33,34] and development of novel, more consistently effective, non-chemical Varroa mite control will likely be long term, sustainable colony management practices. These data demonstrate the complex and dynamic variables that contribute to honey bee colony health. It underscores the importance of controlling for as many of these variables as possible in order to accurately determine the effects of each of these factors as they act alone or in concert with others.

## Figures and Tables

**Table 1:** Honey bee phenothrin bioassay summary. The LD_50_ values are in shown in units of ng phenothrin/mg bee. The LD_50_ values were considered significantly different if the 95% CI of the LD_50_ values did not overlap between colonies or test dates. Letters and numbers beside LD_50_ values indicate significant differences in rows and columns, respectively.

Month	Control			Amitraz			IPM		
	*n*	LD_50_ (95% CI)	Slope (SE)	*n*	LD_50_ (95% CI)	Slope (SE)	*n*	LD_50_ (95% CI)	Slope (SE)
May	308	0.45 (0.35–0.52) ^a123^	2.6 (0.4)	239	0.48 (0.45–0.52) ^a1^	6.4 (0.7)	229	0.47 (0.32–0.57) ^a123^	3.1 (0.6)
June	489	0.45 (0.42–0.46) ^a13^	7.2 (0.5)	576	0.42 (0.40–0.44) ^a2^	7.6 (0.5)	531	0.32 (0.29–0.34) ^b3^	5.8 (0.6)
July	448	0.41 (0.38–0.43) ^a1^	5.3 (0.5)	374	0.43 (0.40–0.48) ^a12^	3.7 (0.5)	329	0.45 (0.41–0.49) ^a1^	5.0 (0.6)
August	383	0.33 (0.29–0.35) ^b2^	4.7 (0.6)	303	0.50 (0.46–0.54) ^a1^	4.8 (0.6)	543	0.35 (0.34–0.38) ^b2^	4.7 (0.4)
September	297	0.48 (0.45–0.51) ^a3^	7.3 (0.7)	252	0.42 (0.39–0.45) ^b12^	6.3 (0.7)	284	0.47 (0.44–0.50) ^ab1^	6.7 (0.7)
October	362	0.41 (0.37-0.44) ^a1^	4.3 (0.6)	464	0.34 (0.31–0.36) ^b3^	4.8 (0.5)	73	0.43 (0.31–0.52) ^ab123^	4.7 (1.4)

**Table 2:** Honey bee amitraz bioassay summary. The LD_50_ values are in shown in units of ng amitraz/mg bee. The LD_50_ values were considered significantly different if the 95% CI of the LD_50_ values did not overlap between colonies or test dates. Letters and numbers beside LD_50_ values indicate significant differences in rows and columns, respectively. The LD_50_ value for the IPM treatment group in October was not reported as the data were not well represented by a line.

Month	Control			Amitraz			IPM		
	*n*	LD_50_ (95% CI)	Slope (SE)	*n*	LD_50_ (95% CI)	Slope (SE)	*n*	LD_50_ (95% CI)	Slope (SE)
May	373	20.0 (18.4–21.8) ^b3^	4.9 (0.4)	339	17.6 (15.8–19.6) ^b5^	3.7 (0.3)	320	27.1 (24.7–29.7) ^a4^	4.2 (0.4)
June	380	59.4 (51.5–72.9) ^a1^	3.4 (0.4)	416	33.7 (31.1–36.6) ^b23^	4.1 (0.4)	368	46.8 (42.0–53.5) ^a12^	3.5 (0.4)
July	429	20.1 (18.5–21.9) ^a3^	4.2 (0.3)	238	19.5 (17.6–21.8) ^a5^	5.0 (0.5)	218	18.4 (16.3–20.9) ^a3^	4.4 (0.5)
August	324	23.8 (19.9–28.5) ^ab3^	2.2 (0.3)	328	25.3 (22.7–28.1) ^a4^	3.5 (0.3)	568	18.5 (16.7–20.5) ^b3^	2.6 (0.2)
September	327	60.6 (55.7–5.8) ^a1^	4.3 (0.5)	180	41.0 (35.8–46.1) ^b123^	4.0 (0.6)	255	36.4 (31.1–41.2) ^b2^	4.0 (0.5)
October	321	30.6 (28.5–32.3) ^b2^	9.1 (1.3)	350	42.4 (38.9–46.3) ^a12^	4.4 (0.5)	NA	NA	NA

**Table 3:** Honey bee clothianidin bioassay summary. The LC_50_ values are in shown in units of ng clothianidin/mL/mg bee. The LC_50_ values were considered significantly different if the 95% CI of the LC_50_ values did not overlap between colonies or test dates. Letters and numbers beside LC_50_ values indicate significant differences in rows and columns, respectively.

Month	Control			Amitraz			IPM		
	*n*	LC_50_ (95% CI)	Slope (SE)	*n*	LC_50_ (95% CI)	Slope (SE)	*n*	LC_50_ (95% CI)	Slope (SE)
May	308	1.12 (0.78–1.43) ^b3^	2.4 (0.3)	269	0.48 (0.09–0.93) ^b4^	1.5 (0.3)	293	2.78 (2.37–3.16) ^a1^	4.0 (0.5)
June	280	0.81 (0.51–1.10) ^a3^	1.9 (0.3)	460	0.35 (0.09–0.64) ^ab4^	1.2 (0.2)	479	0.07 (0.001–0.25) ^b5^	1.0 (0.2)
July	357	1.20 (1.07–1.37) ^a3^	3.3 (0.3)	253	1.14 (1.04–1.28) ^a3^	5.6 (0.7)	276	1.39 (1.22–1.65) ^a34^	3.7 (0.4)
August	325	2.19 (1.95–2.49) ^ab2^	3.7 (0.4)	287	2.47 (2.24–2.71) ^a1^	5.8 (1.0)	416	1.97 (1.81–2.15) ^b23^	4.5 (0.4)
September	370	3.25 (2.94–3.55) ^a1^	4.3 (0.4)	356	2.29 (1.95–2.62) ^b1^	2.8 (0.3)	340	2.59 (2.23–2.97) ^ab1^	2.6 (0.3)
October	296	2.62 (2.33–2.93) ^a2^	4.0 (0.4)	447	1.75 (1.60–1.90) ^b2^	5.3 (0.4)	54	1.60 (0.89–2.04) ^b234^	7.3 (2.2)
